# Molecular mechanisms of cognitive dysfunction following traumatic brain injury

**DOI:** 10.3389/fnagi.2013.00029

**Published:** 2013-07-09

**Authors:** Kendall R. Walker, Giuseppina Tesco

**Affiliations:** Alzheimer's Disease Research Laboratory, Department of Neuroscience, Tufts University School of MedicineBoston, MA, USA

**Keywords:** TBI, cognition, Alzheimer's, CTE, Aβ, tau

## Abstract

Traumatic brain injury (TBI) results in significant disability due to cognitive deficits particularly in attention, learning and memory, and higher-order executive functions. The role of TBI in chronic neurodegeneration and the development of neurodegenerative diseases including Alzheimer's disease (AD), Parkinson's disease (PD), Amyotrophic Lateral Sclerosis (ALS) and most recently chronic traumatic encephalopathy (CTE) is of particular importance. However, despite significant effort very few therapeutic options exist to prevent or reverse cognitive impairment following TBI. In this review, we present experimental evidence of the known secondary injury mechanisms which contribute to neuronal cell loss, axonal injury, and synaptic dysfunction and hence cognitive impairment both acutely and chronically following TBI. In particular we focus on the mechanisms linking TBI to the development of two forms of dementia: AD and CTE. We provide evidence of potential molecular mechanisms involved in modulating Aβ and Tau following TBI and provide evidence of the role of these mechanisms in AD pathology. Additionally we propose a mechanism by which Aβ generated as a direct result of TBI is capable of exacerbating secondary injury mechanisms thereby establishing a neurotoxic cascade that leads to chronic neurodegeneration.

## TBI a global health concern

Traumatic brain injury (TBI) is a leading cause of death and disability in industrialized countries. In the US alone, more than 1.7 million TBI's occur annually and currently, at least 5.3 million Americans are living with a long-term or life-long disability due to a TBI (Faul et al., 2010). The most common causes of TBI in civilian populations are as a result of car accidents, violence (gun and knife wounds) and falls (particularly in the elderly population) (Faul et al., 2010). For those engaged in contact sports, mild TBI's in the form of a concussion (short impairment of brain function) with or without loss of consciousness is a significant risk. While, in military population's blast induced TBI has become the “signature injury” of both the Iraq and Afghanistan conflicts (Levin and Robertson, 2012).

A TBI can be classified as focal in which a direct physical impact to the brain results in the development of a contusion or an extra or sub-dural hemorrhage, or, diffuse in nature where diffuse axonal injury is induced following rapid acceleration/deceleration forces.

Traditionally, a TBI is also classified by severity level based upon a number of criteria including: the level of consciousness, the Glasgow coma scale, and the presence of post-traumatic amnesia (Werner and Engelhard, 2007). Mild TBI which is typically characterized by short-term memory and attention impairments accounts for 75–85% of all TBI's suffered in civilian (Faul et al., 2010) and military populations (Communications). Recently it has become apparent that repetitive mild TBI's, suffered by certain vulnerable populations including athletes involved in contact sports and military personnel can lead to much more significant long-term emotional and cognitive disabilities (Levin and Robertson, 2012).

An overarching consequence of TBI is both short and long-term cognitive deficits. These deficits typically occur in attention, learning and memory and higher order executive functions. Cognitive deficits post-TBI can be attributed to damage to certain vulnerable brain regions including the medial temporal regions, dorso-lateral prefrontal cortex as well as sub-cortical white matter tracts (McAllister, 2011). The hippocampus, which is crucial for declarative memory formation demonstrates atrophy via MRI in a significant proportion of patients who receive a moderate to severe TBI (Bigler et al., 1997). Moderate to severe TBI's are accepted to cause long-term, persistent cognitive deficits in sufferers (Dikmen et al., 2009), while mild TBI is most often associated with short-term cognitive dysfunction that tends to resolve within three months of injury (Levin and Robertson, 2012). However, it is now recognized that a subset of mild TBI patients (up to 15%) develop post-concussion syndrome, this results in persistent cognitive difficulties along with a host of other neurobehavioral symptoms including emotional disturbances and headaches (Levin and Robertson, 2012). Despite significant efforts few therapeutic options exist to prevent or reverse cognitive dysfunction following TBI in humans. Understanding the molecular mechanisms responsible for impaired cognition after TBI will hopefully yield new therapeutic agents.

## Primary and secondary injuries incurred by the brain are responsible for cognitive dysfunction following TBI

The degree of cognitive dysfunction experienced following TBI is determined by the extent of damage to the brain from both the primary and secondary insults/injuries suffered. A direct physical impact to the brain or shear forces (due to rapid angular acceleration/deceleration) from the primary injury causes direct mechanical damage to neuronal and glial cells, the vasculature and strains axons. The damage from the primary injury is immediate and irreversible and as a result is only amenable to preventative protective measures (e.g., personal protective equipment). The primary insult initiates the activation of a vicious cycle of secondary injury effects that include both systemic complications and cellular injury mechanisms that arise over the course of hours to several weeks following the primary injury. Systemic impairments including edema, increased intracranial pressure (ICP) and hemorrhage, all contribute to decreased cerebral blood flow (CBF) and impaired metabolism resulting in ischemia. The ischemia induced by these systemic impairments contributes to the initiation of biochemical and cellular cascades including glutamate excitotoxicity, calcium overload, free radical generation, mitochondrial dysfunction, inflammatory events, and pro-apoptotic gene activation. These cellular injury processes result in neuronal cell loss through necrotic (rapid, uncontrolled) and programmed (delayed) cell death. Programmed cell death can occur by a number of mechanisms including apoptosis (see reviews Zhang et al., 2005; Stoica and Faden, 2010), necroptosis [see reviews (Christofferson and Yuan, 2010; Vandenabeele et al., 2010)] and autophagy [see reviews (Jaeger and Wyss-Coray, 2009; Decuypere et al., 2012)]. The extent of cell loss following TBI has been correlated with cognitive deficits and long-term prognosis in both clinical and experimental studies (see Wakade et al., 2010).

Traditionally, axonal injury was thought to be primarily associated with diffuse injuries. However, it has become apparent that axonal injury in the form of axonal separation is a common sequelae of all TBI's and the extent of axonal injury closely correlates with outcome post-TBI (see Kraus et al., 2007; Johnson et al., 2012a). Improvements in imaging techniques have allowed the detection of extensive white matter tract damage in even mild to moderate cases of TBI. It was originally thought that the majority of axonal separation occurred due to tearing and shearing of the axons during the primary injury. However, it is now known that TBI induced axonal degeneration is as a result of secondary activation of biochemical and cellular injury cascades (Kraus et al., 2007).

**Figure 1:**
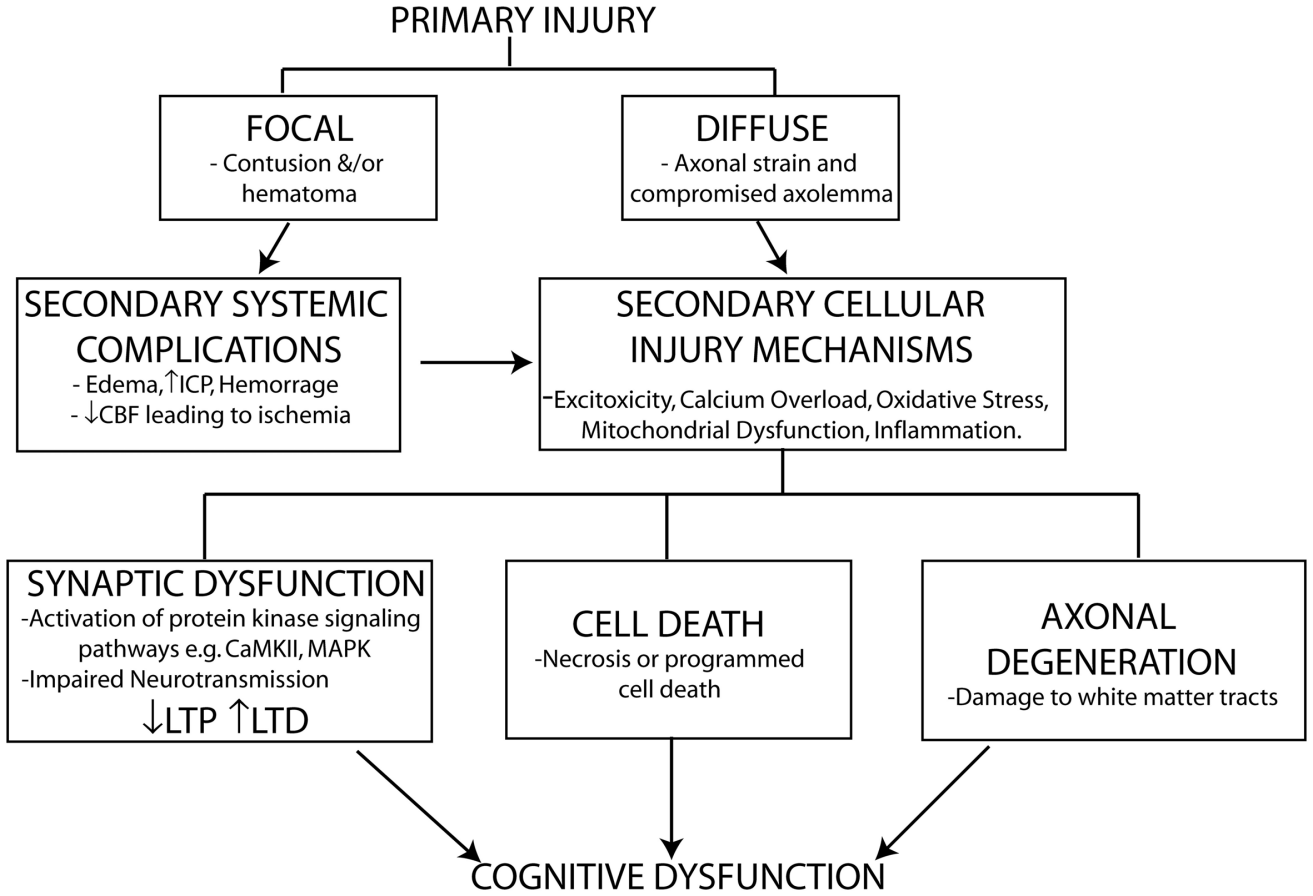
**Schematic representation of primary and secondary phases of injury leading to cognitive dysfunction following TBI.** The primary injury force (direct physical impact or rotational accelaration/decelaration) gives rise to either a focal or diffuse injury which initiates secondary systemic complications and cellular injury mechanisms leading to cell death, axonal injury, and impaired synaptic plasticity contributing to the cognitive dysfunction observed following a TBI. The extent of cell death and axonal injury correlate strongly with neurological outcome following brain injury. TBI induced impairments in synaptic plasticity characterized by impaired long-term potentiation (LTP) and enhanced long-term depression (LTD), two well-known molecular mechanisms controlling memory formation, also contribute to cognitive dysfunction particularly in mild TBI where no overt cell loss is detected despite chronic cognitive deficits being observed.

In addition to neuronal cell loss and axonal degeneration, impaired synaptic plasticity also contributes to cognitive dysfunction particularly in mild to moderate cases of TBI where no overt cell loss is detected. TBI induces abnormalities in neurotransmitter systems (e.g., cholinergic, monoaminergic, and catecholamine), which play important roles in cognition (McAllister, 2011). Impaired calcium homeostasis following TBI affects signaling pathways including those of the protein kinases CaMKII and MAPK, which play important roles in phosphorylating downstream effectors involved in the induction of long-term potentiation (LTP) and long-term depression (LTD), two of the major molecular mechanisms underlying learning and memory (Figure 1).

## Glutamate excitotoxicity and intracellular calcium overload initiate biochemical cascades leading to neuronal death following TBI

Increased extracellular glutamate level is the primary initiating event responsible for intracellular calcium overload and secondary damage following TBI (Arundine and Tymianski, 2004). Increased extracellular glutamate is derived from a number of sources. Mechanical damage from the primary injury compromises cell membranes resulting in the release of glutamate into the extracellular space. Membrane depolarization due to injury-induced ionic imbalances can enhance vesicular release of glutamate. While, injury-induced energy depletion can also lead to a failure in extracellular glutamate uptake by ATP-dependent glial glutamate transporters. Taken together these mechanisms result in dramatically increased extracellular glutamate levels following TBI (Yi and Hazell, 2006).

**Figure 2:**
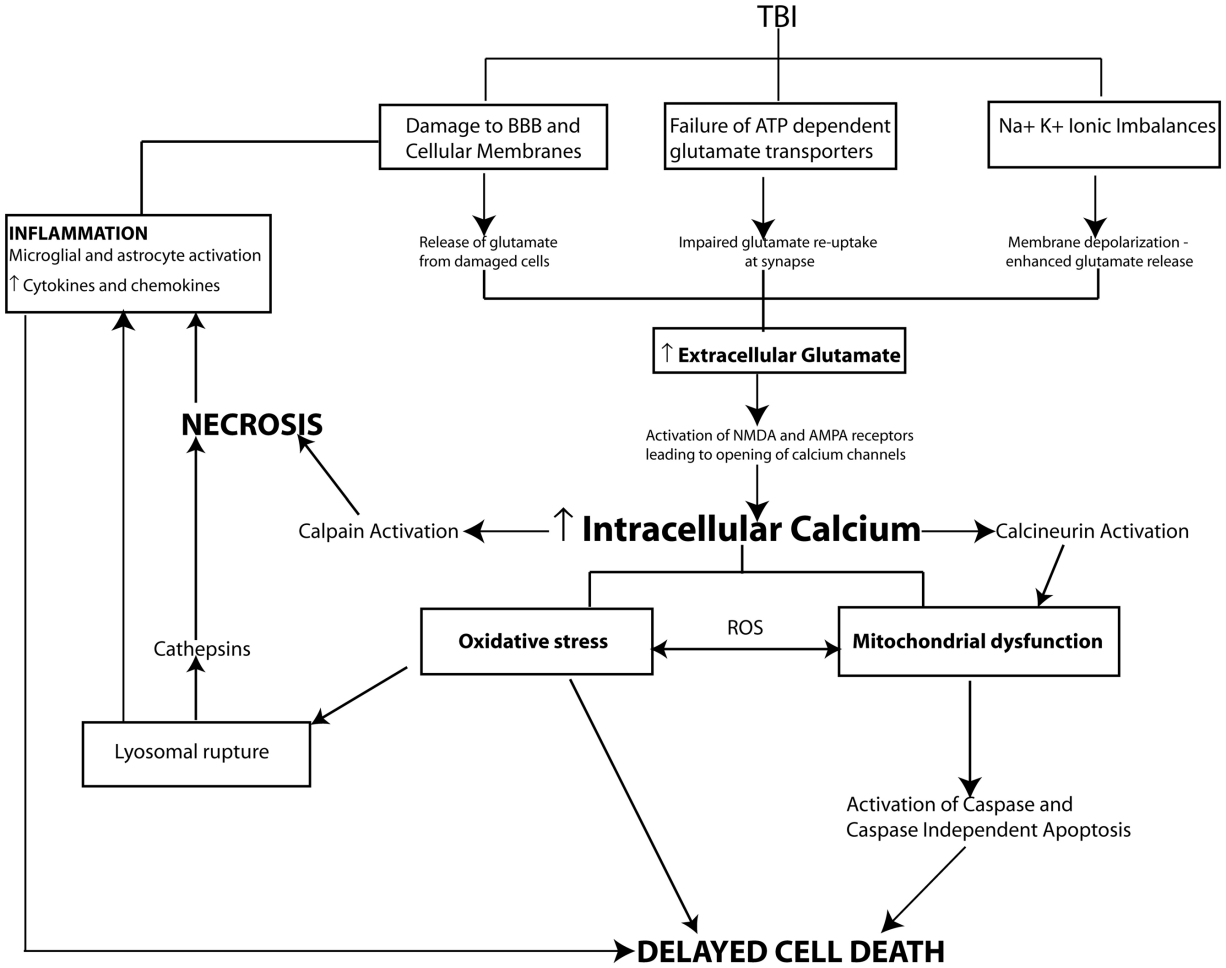
**Pathophysiological mechanisms of cell death following TBI.** In moderate to severe cases of TBI the extent of cell death strongly correlates with cognitive outcome. Following injury cell death can occur by two main pathways, necrosis and programmed cell death. Necrotic cell death is a rapid uncontrolled form of cell loss mediated primarily by calpains and spilled lysosomal cathepsins and contributes to the inflammatory response following TBI. In comparison programmed cell death mechanisms including apoptosis, necroptosis or autophagy occur in a delayed fashion following TBI. A key initiating event in the cell death pathways is TBI induced glutamate excitotoxicity due to damaged cell membranes, enhanced vesicular release of glutamate and impaired glutamate reuptake by glia resulting in increased glutamate levels at the synapse. Increased extracellular glutamate levels activate ionotrophic and metabotropic glutamate receptors leading to intracellular calcium overload and activation of secondary cellular injury mechanisms including calcium sensitive phosphatase and protease activation, mitochondrial oxidative stress, inflammation and proapoptotic gene activation initiating cell death pathways.

Excitotoxicity leads to neuronal injury in two phases. The first phase is characterized by sodium dependent neuronal swelling due to activation of ionotrophic glutamatergic receptors resulting in the opening of sodium channels an influx of Na^+^ ions (and Ca^++^ ions) an efflux of K^+^ ions, which is then followed by delayed calcium dependent neuronal degeneration. The influx of Na^+^ ions leads to membrane depolarization, opening voltage-dependent calcium channels, and removing the magnesium block on NMDA receptors causing a greater influx of calcium into the cytosol (Mark et al., 2001). Calcium influx is further amplified by alterations in AMPA receptor subunit composition (loss of GluR2 subunits) making them more calcium permeable (Luo et al., 2011b). In addition to acting on ionotrophic receptors glutamate can also activate group I metabotropic glutamate receptors thereby stimulating opening of voltage-gated calcium channels and further increasing calcium influx (Niswender and Conn, 2010). This dramatic influx of calcium combined with energy failure initiates the release of intracellular stores of calcium ions (Figure 2).

Excessive intracellular calcium levels trigger the activation of secondary biochemical cascades, which ultimately result in the initiation of programmed cell death and the loss of neurons and synapses in vulnerable brain regions such as the hippocampus producing cognitive dysfunction. Elevated intracellular calcium initiates many cellular pathways including the activation of phospholipases such as calcineurin (CaN), proteases including calpains, and caspases, transcription factors including c-Fos, c-Jun, and c-myc, nitric oxide synthase (NOS) as well as DNA degrading endonucleases. The over-activation or overproduction of any of these molecules can lead to the degradation of cytoskeletal components (e.g., dendritic spine loss), mitochondrial dysfunction, oxidative stress, and pro-apoptotic gene activation (see Ray et al., 2002; Arundine and Tymianski, 2004; Werner and Engelhard, 2007) (Figure 2).

## Modeling cognitive dysfunction and neurodegeneration with experimental TBI

The pathophysiology of TBI observed in humans is extremely heterogeneous in nature and is influenced by a number of parameters associated with the primary injury including the type and severity of trauma and the brain regions affected as well as additional confounders such as age and gender. All of these factors coalesce to initiate the secondary injury processes responsible for cognitive dysfunction and neurodegeneration (Saatman et al., 2008; Margulies et al., 2009). Moreover, the interplay between the cellular injury pathways add an additional element of complexity to identification of molecular mechanisms of cognitive impairment. Hence, experimental animal models of TBI that replicate the human condition are invaluable for the study of these mechanisms.

TBI-induced cognitive dysfunction has been successfully modeled using methods of experimental TBI including weight-drop, rotational/acceleration injury, fluid percussion injury (FPI), controlled cortical impact (CCI) and more recently penetrating ballistic and blast models of TBI (see Albert-Weissenberger and Siren, 2010; Marklund and Hillered, 2011; O'Connor et al., 2011; Xiong et al., 2013). The bulk of research modeling cognitive dysfunction has been carried out in rodents using FPI and CCI models of TBI. Studies have reproducibly demonstrated spatial and working memory deficits, which can persist for more than a year in some cases (Pierce et al., 1998; Dixon et al., 1999; Zohar et al., 2003; Xiong et al., 2013). Rodent TBI models replicate many of the hallmarks of secondary injury identified in human TBI patients including microglial and astrocytic activation along with secretion of pro-inflammatory cytokines and chemokines, axonal injury, glutamate excitotoxicity, cortical spreading depression, mitochondrial dysfunction, free radical generation and neurodegeneration (see Albert-Weissenberger and Siren, 2010).

Experimental TBI in rodents has revealed that neurons within the CA3 region of the hippocampus are particularly vulnerable following injury with studies demonstrating a 60% loss of CA3 pyramidal neurons acutely following injury (Baldwin et al., 1997; Norris and Scheff, 2009). The CA3 pyramidal neurons give rise to the Schaffer collaterals resulting in a partial deaffarentation of the CA1 *regio superior* dendritic field following TBI (Scheff et al., 2005). Studies have shown that the loss of CA3 pyramidal neurons correlates with inhibition of LTP in the CA1 region of the hippocampus following TBI (Scheff et al., 2005). In addition to impairing LTP, LTD is enhanced following TBI furthering impairing learning and memory (Albensi et al., 2000). Interestingly, it has been shown that despite the hippocampus displaying remarkable plasticity with extensive re-innervation occurring following TBI, synapse replacement does not necessarily correlate with an improvement in spatial learning in rodents (Scheff et al., 2005). Taken together with data from studies demonstrating extended alterations in hippocampal synaptic transmission in rodents subjected to TBI (up to 15 days), the prolonged cognitive deficits observed following TBI may be the result of subtle yet chronic alterations in synaptic plasticity beyond overt neuronal loss (Miyazaki et al., 1992; Reeves et al., 1995; D'Ambrosio et al., 1998; Sick et al., 1998; Albensi et al., 2000; Sanders et al., 2000; Witgen et al., 2005; Wakade et al., 2010; Zhang et al., 2011). Activation of calcium dependent phosphatases (calcineurin) and proteases (calpains) following TBI have been demonstrated to impair neurotransmission by mediating the retraction and collapse of dendritic spines, ultimately leading to spine loss and cognitive deficits (Posmantur et al., 1997; Saatman et al., 2010; Gao et al., 2011; Campbell et al., 2012a). Additionally, TBI has been shown to induce abnormalities in a number of neurotransmitter systems, which play important roles modulating cognition. The random activation of glutamate receptors following TBI is a fundamental element of the injury process. Following the initial flood of extracellular glutamate and the resultant hyperexcitability there is a delayed downregulation of glutamate receptors that can last for many days in experimental models (see reviews Arundine and Tymianski, 2004; Luo et al., 2011b). Such alterations in glutamatergic signaling are implicated in impaired induction of LTP and enhanced induction of LTD following TBI. The reduction in inhibition along with the hyperexcitability observed in the hippocampus following TBI is evidence of impaired GABAergic neurotransmission. Studies have demonstrated reduced GABA receptor binding and alterations in GABA receptor subunit composition following experimental TBI (Gibson et al., 2010). Importantly, impaired GABAergic signaling has been implicated the development of epileptogenic events post-injury (see review Imbrosci and Mittmann, 2011). TBI has also been implicated in the chronic impairment of cholinergic neurotransmission. Following injury a massive release of Acetylcholine (ACh) occurs resulting in a downregulation in muscarinic acetycholine receptor binding affinity, which is then followed by a prolonged depression in ACh release. Studies have shown that the ability of the cholinergic system to respond to an evoked release of ACh is chronically impaired following experimental cortical injury (see review Tenovuo, 2006). Additionally, alterations in dopaminergic signaling have been identified in the prefrontal cortex and striatum of rodents following experimental TBI. These alterations include changes in tissue levels of dopamine and the dopamine transporter, along with acute increases in the dopamine D1 receptor level along with delayed increases in tyrosine hydroxylase synthesis, the rate-limiting enzyme involved in catecholamine synthesis (see review Bales et al., 2009).

## Attenuating secondary injury biochemical cascades confers neuroprotection and improves cognitive outcome following experimental TBI

Therapeutic strategies to prevent the spatial memory deficits observed in rodents following experimental TBI have focused on intervening in the secondary injury cascade to prevent neuronal loss, axonal injury and improve synaptic plasticity. A primary initiating event in this cascade is glutamate-mediated excitotoxicity. It is mediated primarily through abnormal activation of the highly calcium permeable NMDA receptors resulting in intracellular calcium overload and induction of neurotoxic cascades. As a result, therapeutic interventions have focused on antagonizing NMDA and to a lesser extent AMPA receptors in animal models of TBI. Pharmacological blockade using MK-801 (Phillips et al., 1998; Biegon et al., 2004; Han et al., 2009), CP-89-113 (Okiyama et al., 1998), memantine (Rao et al., 2001) and telampenal (Belayev et al., 2001) resulted in neuroprotection and improved cognition following experimental TBI. However, due to the important role glutamate plays in normal excitatory signaling this therapeutic strategy has largely failed in human clinical trials. Recently, Feng et al. (2011) described an alternate strategy to prevent glutamate-mediated excitotoxicity by targeting glutamate release rather than antagonizing glutamatergic receptors. They demonstrated that reducing extracellular glutamate levels using the *N*-acetylaspartylglutmate (NAAG) peptidase inhibitor (ZJ-43) greatly improved cognitive performance following experimental TBI in rats by preventing the hydrolysis of the NAAG peptide neurotransmitter, which regulates synaptic release of glutamate (Feng et al., 2011). This strategy augments an endogenous control mechanism for glutamate release but requires further investigation to determine if it demonstrates advantages over glutamatergic receptor antagonism.

Mitochondrial dysfunction also plays an important role in cognitive dysfunction following TBI. Deficits in mitochondrial bioenergetics occur rapidly following experimental TBI (<1 h) and can persist for up to 14 days (Xiong et al., 1997; Gilmer et al., 2009). The significant structural and functional damage mitochondria undergo following TBI is mediated primarily by glutamate-mediated excitotoxicity resulting in mitochondrial calcium overload. Following trauma, excessive intracellular calcium can exhaust the calcium buffering ability of mitochondria leading to the formation of the mitochondrial permeability transport pore (mPTP). A consequence of mPTP formation is the loss of membrane potential resulting in an uncoupling of electron transport from ATP production and the release of pro apoptotic factors (cytochrome c and AIF) and activation of cell death pathways (see Cheng et al., 2012). A number of studies have shown that blocking key components of mPTP formation in rodent experimental models of TBI using cyclosporin A and cyclosporin analogues such as NIM811 dramatically improves mitochondrial function and cognitive performance (Scheff and Sullivan, 1999; Sullivan et al., 1999; Readnower et al., 2011).

Additionally, mPTP formation results in the production of reactive oxygen species (ROS) generating free radicals and causing oxidative damage to cells. Oxidative damage plays an important role in cell death pathway activation, axonal injury and impaired synaptic plasticity leading to cognitive deficits post-TBI (Wu et al., 2006; Ohta et al., 2013) (see Cheng et al., 2012). ROS generated by dysfunctional mitochondria and reactive nitrogen species generated through glutamate-mediated NMDAR activation can form the highly reactive species peroxynitrite, which is toxic to cells. Peroxynitrite induces protein nitration, lipid peroxidation and DNA fragmentation activating caspase independent apoptotic cell death (Ansari et al., 2008). A number of studies have demonstrated that attenuating oxidative stress can improve cognitive function following TBI (Wu et al., 2004, 2006; Hou et al., 2012; Ohta et al., 2013).

TBI-induced neuroinflammation is a highly complex process with both neuroprotective and neurotoxic components. Following TBI, astrocytes and microglia resident in the CNS respond to the disruption of the blood brain barrier (BBB) becoming activated. Activated glia secretes a range of proinflammatory cytokines and chemokines that are neurotoxic. Levels of the proinflammatory cytokines Tumor necrosis factor (TNF), Interleukin 1β (IL-1β) and Interleukin-6 (IL-6) have been shown to increase dramatically within hours of a TBI (Lloyd et al., 2008). Binding of cytokine receptors activates numerous intracellular signaling pathways including c-Jun N-terminal kinase (JNK), p38 mitogen activated protein kinase (p38/MAPK) and extracellular signaling-related kinase (ERK) which play key roles in synaptic plasticity (Pearson et al., 2001). Furthermore, cytokine binding can promote cell death through the activation of caspases (Wallach, 1997). In addition to secreting cytokines microglia have been demonstrated to secrete additional substances including ROS and nitrogen species along with glutamate, which under normal conditions play an important role in neural transmission but when microglia become chronically activated under inflammatory conditions further contributes to neuronal cell death (see review Block et al., 2007). The neuroinflammatory response initiated following TBI is highly persistent with inflammation detected for at least one year post-injury in animal models and many years in humans (Ramlackhansingh et al., 2011; Acosta et al., 2013). Neuroinflammation and chronic microglial activation have been shown to play an important role in cognitive dysfunction post-TBI. Importantly, the persistence of the inflammatory response following TBI provides an important therapeutic window in order to prevent neurodegeneration. Administration of compounds that prevent microglial activation (Sauerbeck et al., 2011; James et al., 2012) and/or suppress the release of proinflammatory cytokines and chemokines (Lloyd et al., 2008; Clausen et al., 2009) have been demonstrated to dramatically improve long-term cognitive function following experimental TBI.

An endpoint of these biochemical cascades is the induction of programmed cell death. Neuronal loss has been demonstrated to correlate with cognitive deficits and prognosis both clinically and experimentally following TBI (see Wakade et al., 2010). Two phases of neuronal cell death occur following TBI. Immediately following the primary injury, necrosis, an uncontrolled passive form of cell death occurs, which, is then followed by a delayed wave of programmed cell death. Necrotic cell death is mediated by calpains and spilled lysosomal cathepsins and contributes to the inflammatory response witnessed following TBI (Artal-Sanz and Tavernarakis, 2005; McAllister, 2011). While the cell death that occurs immediately following the primary injury through mechanical damage and rapid activation of necrosis is not amenable to therapeutic intervention, the delayed nature of programmed cell death provides an important therapeutic window. Strategies aimed directly at inhibiting apoptosis (Knoblach et al., 2002; Abrahamson et al., 2006; Bao et al., 2011; Li et al., 2011a; Piao et al., 2012), necroptosis (You et al., 2008) and autophagy (Luo et al., 2011a) have been shown to be neuroprotective following experimental TBI reducing cell loss and improving outcome including motor function and spatial memory. Evidence suggests that following TBI multiple cell death pathways may be activated independently and function in parallel. Additionally, there may be cross talk between cell death pathways (Piao et al., 2012; Wang et al., 2012). In light of this, Piao et al., 2012 recently published a study demonstrating that therapeutic targeting of multiple cell death pathways, in this case both caspase-dependent and independent forms of apoptosis provides superior neuroprotection than those aimed only at a single pathway (Piao et al., 2012).

In order to translate these experimental successes into clinically relevant therapies to ameliorate cognitive dysfunction following TBI, we must understand the underlying molecular pathways that mediate these secondary injury cascades.

## TBI, the aging brain and cognitive dysfunction

The aged brain is much more vulnerable to the effects of TBI and outcome is much worse in older subjects who receive a brain injury. There is accumulating evidence that TBI accelerates the onset of cognitive decline leading to dementia (Sullivan et al., 1987; Gedye et al., 1989; Schofield et al., 1997; Nemetz et al., 1999). Aging is one of the most significant risk factors for the development of dementia of the Alzheimer's Type. It is possible that TBI combines with normal ageing processes resulting in the exacerbated cognitive decline witnessed in some people who suffered a TBI (Moretti et al., 2012). Experimental studies have replicated the greatly impaired functional and cognitive outcome observed in aged humans following TBI using aged rodents (Onyszchuk et al., 2008; Sandhir et al., 2008). Interestingly, calcium homeostasis is significantly more dysregulated in the hippocampus of aged animals than younger animals following TBI which could lead to prolonged activation of secondary injury cascades and increased cell death (Osteen et al., 2001). In keeping with this theory studies have demonstrated exacerbated inflammatory responses including exaggerated microglial and astrocytic activation associated with increased numbers of dying neurons; higher levels of oxidative damage, reduced expression of neuroprotective genes, as well as increased mitochondrial dysfunction, particularly in the synapses of aged rodents following brain injury (Sandhir et al., 2004, 2008; Shah et al., 2006; Shao et al., 2006; Anderson et al., 2009; Gilmer et al., 2010; Itoh et al., 2013). Additionally, signaling pathways crucial to the formation of LTP are significantly more impaired in older animals post-TBI. Moderate to severe experimental TBI is known to impair the cAMP-PKA pathway which plays a key role in the induction of LTP (Titus et al., 2013). However, a recent study demonstrated that in aged animals even mild TBI is sufficient to cause significant decreases in cAMP levels in the hippocampus but not in young animals. This reduction in cAMP coincides with reduced CA1 hippocampal LTP, which is partially reversable with Rolipram, a drug which increases cAMP levels (Titus et al., 2013). Analysis of the effects of TBI on aged animals may explain age related deficits in synaptic plasticity and the reason why older patients demonstrate prolonged cognitive impairment following mild TBI compared to younger patients.

## Traumatic brain injury and chronic neurodegeneration

It is generally accepted that TBI can precipitate on-going processes/cascades and *in vivo* conditions that lead to chronic neurodegeneration and the development of neurodegenerative diseases such as Parkinson's disease (PD), Alzheimer's disease (AD), Amyotrophic lateral sclerosis (ALS), and the newly classified entity chronic traumatic encephalopathy (CTE) (see Uryu et al., 2007; Lehman et al., 2012). The potential for head trauma to precipitate conditions amenable to chronic neurodegeneration is not a new phenomenon. It has been well known that repetitive head trauma could lead to cognitive decline since the phenomenon of *dementia pugilistica* (DP) or “punch–drunk” syndrome was first described in boxers in 1928 (Martland, 1928). Today DP is refered to as a variant of CTE due to the accumulation of hyperphophorylated tau in the brain and is a classic example of repetitive mild TBI leading to dementia later in life. Evidence from histopathological and biochemical analysis of biopsy material from TBI patients and sufferers of neurodegenerative disorders indicates that the molecular pathways of TBI induced neurodegeneration and those of neurodegenerative disease must overlap. Material obtained from both conditions show remarkably similar pathology and accumulation of identical proteins implicated in disease pathogenesis including amyloid precursor protein (APP), β-secretase (BACE1), presenilin 1 (PS-1), tau and α-synuclein (Chen et al., 2004; Uryu et al., 2007; Sivanandam and Thakur, 2012).

In recent years there has been increased media attention on the long-term effects of brain trauma. This has primarily focused on trauma in form of concussion suffered by certain vulnerable populations such as athletes engaged in contact sports. A recent study of National Football League (NFL) players demonstrated that football players have neurodegenerative mortality rates three times higher than the general US population and four times the national rate for the neurodegenerative sub-categories of AD and ALS (Lehman et al., 2012). The risk of cognitive impairment appears to increase with the number of insults suffered, with one study demonstrating a 5-fold increase in mild cognitive disorders and a 3-fold prevalence of significant memory problems in players who suffered three or more concussions (Guskiewicz et al., 2005). While the case for repetitive insults causing chronic neurodegeneration and cognitive impairment is solid, there is increasing evidence that a single moderate to severe head trauma is sufficient to cause chronic neurodegeneration and the development of a neurodegenerative disorder such as AD in some individuals (Mortimer et al., 1985, 1991; Graves et al., 1990; Molgaard et al., 1990; O'Meara et al., 1997; Salib and Hillier, 1997; Schofield et al., 1997; Guo et al., 2000; Plassman et al., 2000; Fleminger et al., 2003; Johnson et al., 2012b).

The molecular mechanisms responsible for chronic neurodegeneration, cognitive impairment, and increasing a persons risk of developing a neurodegenerative disorder following a TBI remain largely unknown. It is apparent that single moderate to severe episodes of head trauma appear to be associated with the pathological accumulation of the Aβ peptide, while mild repetitive traumas are associated with increased tau phosphorylation (Dekosky et al., 2010). In this review we will focus on experimental evidence of potential molecular mechanisms that have been uncovered linking TBI to the development of two forms of dementia: AD (characteristic Aβ and tau pathology) and CTE (a tauopathy).

## TBI-induced AD pathology

AD is the one of the most common neurodegenerative diseases, and accounts for approximately 70% of all cases of dementia. The key pathological hallmarks of AD are extracellular senile plaques composed of neurotoxic Aβ peptide and intracellular neurofibrillary tangles (NFT) composed of hyperphosphorylated tau. Aβ is a 38–43 amino acid peptide that is generated by the sequential cleavage of the amyloid precursor protein (APP) by β-secretase (BACE1) and γ-secretase enzymes. Tau is a microtubule-associated protein that under normal conditions plays an important role in stabilizing axonal microtubules. While it is clear that both Aβ and tau play important roles in AD pathology, under the amyloid cascade hypothesis it is generally accepted that Aβ generation and amyloid plaque deposition are the initiating events in the pathology of AD and precede NFT formation. AD is a highly complex disease, the vast majority of cases are sporadic and most likely arise from the complex interactions of multiple genes with each other as well as the environment (Reitz et al., 2011). TBI is one of the strongest environmental risk factors for the development of AD and there is significant evidence that a single event of moderate to severe TBI is sufficient to increase the risk of developing AD. A pathological link between TBI and the risk of later development of AD was established by the demonstration of Aβ plaques and intra-axonal accumulation of Aβ in 30% of patients who died acutely after suffering a severe TBI (Roberts et al., 1991, 1994; Gentleman et al., 1997; Smith et al., 2003a,b; Ikonomovic et al., 2004; Dekosky et al., 2007; Uryu et al., 2007; Chen et al., 2009). Plaque deposition following TBI occurred regardless of age as they were detected in children. However, in comparison to AD in which senile plaques develop slowly, the plaques observed in TBI patients developed rapidly often within 2 h of injury (Roberts et al., 1991, 1994). These observed plaques were diffuse in nature similar to very early stage AD rather than the dense neuritic plaques observed in late stage AD. This is despite the fact that they were predominately composed of Aβ_42_, the aggregation prone form of Aβ (Dekosky et al., 2007). Interestingly the appearance of plaques in TBI patients appears to be temporally regulated, with plaques disappearing in the months following injury (Chen et al., 2009). Whereas, analysis of brains from TBI patients many years post injury reveals plaques in greater numbers than in age-matched controls (Johnson et al., 2012b). Interestingly, in keeping with the amyloid hypothesis, the presence of NFT's is not observed acutely following TBI, but is observed in patients who survive many years following a single severe episode of TBI (Johnson et al., 2012b).

Soluble Aβ levels are increased and Aβ deposition occurs rapidly both clinically and experimentally following TBI (Johnson et al., 2010). Increased levels of the key components of amyloidogenic cascade APP, BACE1, and PS-1 have been identified in the damaged axons from human TBI patients (Uryu et al., 2007; Chen et al., 2009). Enhanced APP immunoreactivity is used as a diagnostic marker for axonal damage post-TBI. The intra-axonal accumulation of Aβ in white matter does not fit with the typical picture of Aβ generation in AD which is preferentially limited to the gray matter. Chen and colleagues proposed a model to explain the increased synthesis of Aβ following TBI induced axonal damage. They proposed that the disruption of axonal transport that occurs following injury results in the accumulation in axonal bulbs of APP and the APP-secretases responsible for its processing into Aβ thereby facilitating the increased generation of Aβ. Following lysis and axonal breakdown this Aβ could be released into the brain parenchyma where it could be sequestered into plaques (Chen et al., 2004). Aβ accumulation in axonal bulbs has since been demonstrated by other researchers in both humans and animals following trauma (see Johnson et al., 2012a). It should be noted that while increases in APP immunoreactivity in damaged axons following TBI has been demonstrated clinically and experimentally, whether this represents an overall increase in total APP levels in tissue is subject to debate. We and others failed to detect an increase in total APP levels in tissue by western blotting in the acute phase post-injury, it may be that while APP concentrations increase in axonal varicosities, the total pool in tissue does not (Ciallella et al., 2002; Abrahamson et al., 2006; Schwetye et al., 2010; Walker et al., 2012). Alternatively these discrepancies could arise due to differences in experimental TBI protocols (Table 1).

**Table 1:** **Common pathological features in TBI and AD**.

**Pathology**	**TBI (clinical and experimental)**	**AD (human brain and transgenic mouse model)**
↑Soluble Aβ and Aβ plaque deposition	Walker et al., 2012; see review Johnson et al., 2010	See review Torres-Aleman, 2008; Perl, 2010
↑Tau-P and NFT's	Tran et al., 2011; Johnson et al., 2012b	See review Torres-Aleman, 2008; Perl, 2010
Neuronal cell loss	Baldwin et al., 1997; Wakade et al., 2010	See review Torres-Aleman, 2008; Perl, 2010
Synapse loss	Wakade et al., 2010; Gao et al., 2011	Reddy and Beal, 2008; see review Arendt, 2009
Dendritic spine loss and remodeling	Gao et al., 2011; Campbell et al., 2012a	Knobloch and Mansuy, 2008; Hudry et al., 2012

## Aβ genesis post-TBI: a role for BACE1

BACE1 is the key rate-limiting enzyme involved in the generation of Aβ. BACE1 is a stress related protein and levels are elevated in the brains of AD patients (Cole and Vassar, 2008). We and others have demonstrated that BACE1 levels are dramatically increased following experimental TBI and this increase coincides with elevated Aβ production (Blasko et al., 2004; Loane et al., 2009; Walker et al., 2012). Inhibition of BACE1 is therapeutic post-TBI in rodents (Loane et al., 2009) and is being actively pursued as a therapeutic strategy for the treatment of AD. BACE1 levels and activity are regulated at many levels within the cell including, at the transcriptional, translational and post-translational levels (see Walker et al., 2012). We have recently identified a novel molecular mechanism involving the post-translational regulation of BACE1 by the adaptor protein GGA3. We have previously shown that the adaptor protein GGA3 plays a key role in trafficking BACE1 from the endosomes to the lysosomes for degradation (Tesco et al., 2007; Kang et al., 2010). Our studies demonstrate that GGA3 is capable of regulating BACE1 levels both *in vitro* and *in vivo* and that impaired degradation of BACE1 due to caspase-3 cleavage of GGA3 results in dramatically increased Aβ levels *in vitro* (Tesco et al., 2007; Walker et al., 2012). GGA3 levels are reduced by 50% in the temporal cortex of AD patients concurrently with caspase-3 activation, indicating that reduced levels of GGA3 may play an important role in regulating BACE1 levels in the brains of AD patients (Tesco et al., 2007). We have recently demonstrated that caspase-3 mediated cleavage of GGA3 and its closely related homolog GGA1, synergistically regulate BACE1 levels and Aβ production in the acute phase (48 h) following experimental TBI in rodents. Importantly, using GGA3+/− mice we have demonstrated that a 50% reduction in GGA3 (as has been identified in the brains of AD patients) is sufficient to cause a sustained elevation of BACE1 levels and activity and Aβ production in sub-acute phase (7days) of injury when GGA1 levels have returned to normal. This is the first demonstration of a molecular mechanism of BACE1 elevation following TBI. Taken together with our data that demonstrates that GGA3 plays a key role in regulating BACE1 levels *in vivo*, impaired degradation of BACE1 due to reduced levels of GGA3 represents an attractive molecular mechanism linking acute brain injury to chronic Aβ production and neurodegeneration. As Aβ has been shown to be able to stimulate caspase-3 activation (Mattson et al., 1998; Kasa et al., 2004), we postulate that TBI induced Aβ would be expected to result in further caspase activation leading to additional depletion of GGA1 and GGA3 and increased elevation of BACE1 and Aβ which could create a vicious self perpetuating cycle. This data supports the hypothesis that subjects with lower levels of GGA3 may be at greater risk of developing AD following TBI.

## Aβ clearance mechanisms post-TBI

Aβ clearance mechanisms also play an important role in the overall level of Aβ in the brain witnessed following TBI and in the brains of AD patients. In addition to microglial activation a number of other naturally occurring mechanisms exist to clear Aβ from the brain. Neprilysin, a zinc-metalloendopeptidase is the primary scavenger of Aβ *in vivo* and has been shown to be upregulated following TBI. Neprilysin immunoreactivity is upregulated for up to 3 years following TBI presumably as a neuroprotective mechanism. This enhanced neprilysin immunoreactivity observed in long-term survivors of TBI was closely associated with the absence of Aβ plaques (Chen et al., 2009). The importance of neprilysin in Aβ clearance has been demonstrated by Iwata and coworkers who showed that endogenous Aβ increased in a dose-dependent manner in neprilysin null mice (Iwata et al., 2001). Importantly, neprilysin has been implicated in AD pathogenesis with decreased levels of neprilysin observed in the brains of AD patients (Yasojima et al., 2001a,b; Caccamo et al., 2005). Johnson and colleagues identified a polymorphism in the promoter region of the neprilysin gene that correlated with Aβ deposition after TBI (Johnson et al., 2009). However, the role of neprilysin polymorphisms in Aβ pathogenesis following TBI and in AD requires further investigation.

APOε is the major lipid carrier in the brain and it has been shown to be increased both clinically and experimentally following TBI, presumably to scavenge lipids released following cell death and myelin breakdown (Laskowitz et al., 1998; Iwata et al., 2005). Importantly, APOε has been shown to regulate Aβ metabolism and it has been theorized that improving APOε function may facilitate neuronal repair and increase Aβ clearance post-TBI. Lipidated APOε binds Aβ and delivers it to microglia for degradation by neprilysin. The more richly lipidated APOε is, the greater the clearance of Aβ (Fitz et al., 2010). The ATP-binding cassette transporter A1 (ABCA1) plays a key role in Aβ clearance. ABCA1 regulates cholesterol and phospholipid efflux from cells to lipid poor apolipoproteins APO A-I (plasma) and APOε (CNS) to generate high-density lipoproteins (HDL) (Brooks-Wilson et al., 1999). APP transgenic mice with only one functional copy of ABCA1 display significant cognitive deficits, which correlate with the level of soluble Aβ (Lefterov et al., 2009). Conversely increasing ABCA1's expression pharmacologically or genetically significantly ameliorates amyloid pathology and improves cognitive function in transgenic mice (see Fitz et al., 2012). A number of studies have demonstrated that upregulation of ABCA1 using LXR agonists is neuroprotective improving cognitive outcome following experimental TBI in both transgenic and non-transgenic mice (Loane et al., 2011; Namjoshi et al., 2013). In humans, the APOε4 allele has been shown to be associated with increased risk of developing AD, while the APOε2 allele appears to offer some risk protection. While, the role of APOε4 in modulating outcome following TBI appears to be much more complex, it is generally accepted that the presence of the APOε4 allele confers poor neurological outcome following TBI (see Verghese et al., 2011). A recent study by Fitz et al. (2012) demonstrated the role of ABCA1 in modulating APOε level in the presence of the APOε4 allele, which is associated with increased AD risk. The APOε4 background resulted in increased deposition of Aβ and impaired cognitive function concurrent with a reduction in APOε and APOA-1 when ABCA1 was depleted by 50%, but not when the APOε3 allele was present which is not associated with increased AD risk (Fitz et al., 2012). The reduction of APOε witnessed in ABCA1 null mice is hypothesized to be due to its increased metabolism due to poor lipidation and stability, as mRNA levels were unaffected (Wahrle et al., 2004). This study highlights an important link between APOε4 status and reduced Aβ clearance following TBI representing a potential mechanism leading to chronic neurodegeneration.

**Figure 3:**
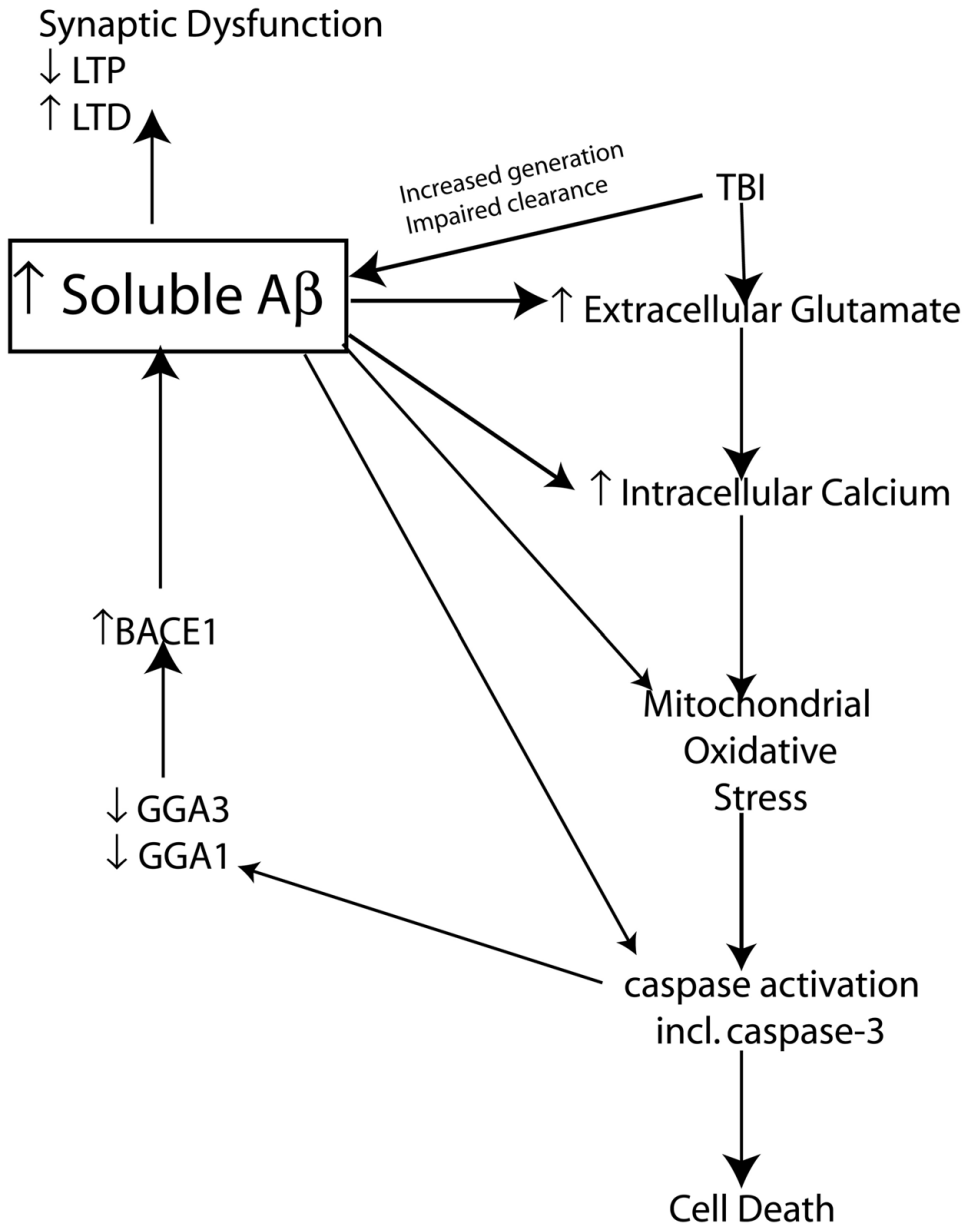
**Schematic representation of the proposed mechanism by which TBI induced increases in Aβ create a vicious neurotoxic cascade leading to chronic neurodegeneration.** Soluble Aβ levels are increased both clinically and experimentally following TBI and are known to impair synaptic plasticity and hence cognitive function by inhibiting LTP and enhancing LTD mechanisms of memory formation. The increased Aβ observed following TBI presumably occurs through a combination of increased generation and impaired clearance mechanisms. BACE1 levels are elevated following experimental TBI and directly contribute to the observed increases in Aβ. Caspase-3 mediated cleavage of BACE1 interacting adaptor proteins GGA1 and GGA3 has been demonstrated to modulate BACE1 levels and Aβ production following experimental TBI. The increased Aβ levels observed due to mechanisms including caspase mediated depletion of GGA1 and GGA3 could theoretically create a vicious feed forward mechanism leading to further propagation of Aβ. *In vitro* evidence suggests that Aβ is capable of initiating and mediating many of the cellular injury mechanisms that lead to programmed cell death following TBI.

It is clear that soluble Aβ levels are elevated clinically and experimentally following TBI through an interplay of generation and clearance mechanisms. It is well known that soluble Aβ can mediate a variety of neurotoxic functions that could hypothetically be responsible for creating “feed forward” mechanisms that following its initial increase as a result of TBI could establish a vicious neurotoxic cascade ultimately leading to chronic neurodegeneration. This neurotoxic cascade could provide the link between Aβ increases following acute brain injury and the development of AD. In the following sections we will review experimental evidence of potential mechanisms that modulate Aβ and tau following TBI and provide evidence of the role of these mechanisms in AD pathology. We will provide evidence that Aβ itself is capable of mediating many of these mechanisms and maybe the crucial factor leading to chronic neurodegeneration following brain injury (Figure 3).

## Synaptic dysfunction and loss closely correlates with cognitive impairment following TBI and in AD brain

In addition to overt neuronal loss, synaptic dysfunction and synapse loss are pathophysiological hallmarks of both TBI and AD. Synaptic dysfunction/degeneration is considered one of the most reliable markers of cognitive impairment in AD and can be detected very early on in the disease process, as early as MCI, the pro-dromal state of AD (Arendt, 2009). Studies have demonstrated an 18% loss of synapses in the CA1 hippocampal region of MCI patients, which progressed to a 55% loss in mild AD cases. The progression of AD is marked by a progressive loss of synapses eventually accompanied by a 10–20% loss of cortical neurons (see Arendt, 2009). In AD, synapse loss is not always confined to dying neurons but can also occur in surviving neurons (Coleman and Yao, 2003). Analysis of synaptic components from brains of AD patients revealed decreased levels of pre-synaptic (as measured by synaptophysin) and post-synaptic (as measured by synaptopodin and PSD-95) proteins. Importantly, synaptic loss appears to be confined to brain regions affected by AD and closely correlates with NFT counts indicating a link between synaptic dysfunction and tangle formation in AD patients (see Reddy and Beal, 2008; Arendt, 2009).

Synaptic loss as measured by decreased levels of synaptophysin and PSD-95 has been demonstrated in both immature and mature rodents following TBI (Gobbel et al., 2007; Ding et al., 2009; Wakade et al., 2010; Gao et al., 2011). The cognitive impairment observed was closely associated with the degree of synaptic loss and recovery of cognitive performance following TBI correlated with increased levels of synaptic marker proteins. PSD-95 is a scaffolding protein that plays an important role in synaptic maturation and plasticity associated with NMDA receptor signaling. It has been demonstrated to control activity dependent AMPA receptor incorporation at the synapse during LTP. Due to its important role in maintaining synaptic plasticity and strengthening LTP induction, its loss in the hippocampus has been implicated in the cognitive deficits observed following TBI and in AD (see Gobbel et al., 2007 and review Proctor et al., 2011).

Upregulation of members of the matrix metalloproteinase (MMP) family, in particular MMP-9 a potent inflammatory mediator, via HIF1α has been implicated in the loss of synapses witnessed post-TBI. TBI-induced expression of HIF-1α leads to synapse loss that closely correlates with the upregulation of MMP-2 and MMP-9. Synaptic loss was prevented by inhibiting HIF1α expression and MMP-9 upregulation by the administration of the anti-inflammatory drug minocycline (Ding et al., 2009). MMP's are a family of proteins that are involved in regulating extracellular matrix proteins and under normal conditions play an important role in synaptic plasticity and cognition. MMP-9 levels have been demonstrated to increase concordantly with increasing injury severity and play an important role in edema formation following experimental TBI (see Copin et al., 2012). Additionally, increased MMP-9 plasma levels are an important predictor of length of hospital stay and death in severe TBI patients (see Copin et al., 2012). The importance of MMP-9 to outcome following TBI is reinforced by data demonstrating that MMP-9 knockout mice suffer greatly reduced tissue and cell loss and improved functional outcome following TBI (Wang et al., 2000). The role of inflammation-induced increases of MMP-9 in the synaptic dysfunction witnessed in AD is of particular interest as MMP-9 levels are elevated in the plasma and brain of AD patients (Lorenzl et al., 2003) and neuroinflammation is a feature of the AD brain.

Aβ and in particular oligomeric Aβ species have been demonstrated to play an important role in impairing LTP and modulating synapse density and hence cognition in a variety of experimental settings including hippocampal slice cultures and transgenic mouse models (see Revett et al., 2013). Elevated oligomeric Aβ levels in the hippocampus and cortex which occurs during the progression of AD and following head injury could play a role in mediating cognitive decline through disruption of the excitatory glutamatergic synapses.

## Glutamate and Aβ-mediated excitotoxicity following TBI and in AD brains

Excessive NMDA receptor activation leading to excitotoxicity has been implicated in the pathophysiology of both TBI and AD. Chronic stimulation of NMDA receptors by glutamate or NMDA has been demonstrated to alter APP processing and stimulate Aβ production in neurons presumably through impaired calcium homeostasis (Willoughby et al., 1995; Lesne et al., 2005). This is supported by a strong correlation between Aβ level and neuronal firing at excitatory synapses under normal conditions and following TBI. This relationship is particularly evident in brain regions vulnerable to Aβ deposition in AD mouse models (Buckner et al., 2005; Cirrito et al., 2005, 2008; Brody et al., 2008; Marklund et al., 2009; Schwetye et al., 2010; Bero et al., 2011).

**Figure 4:**
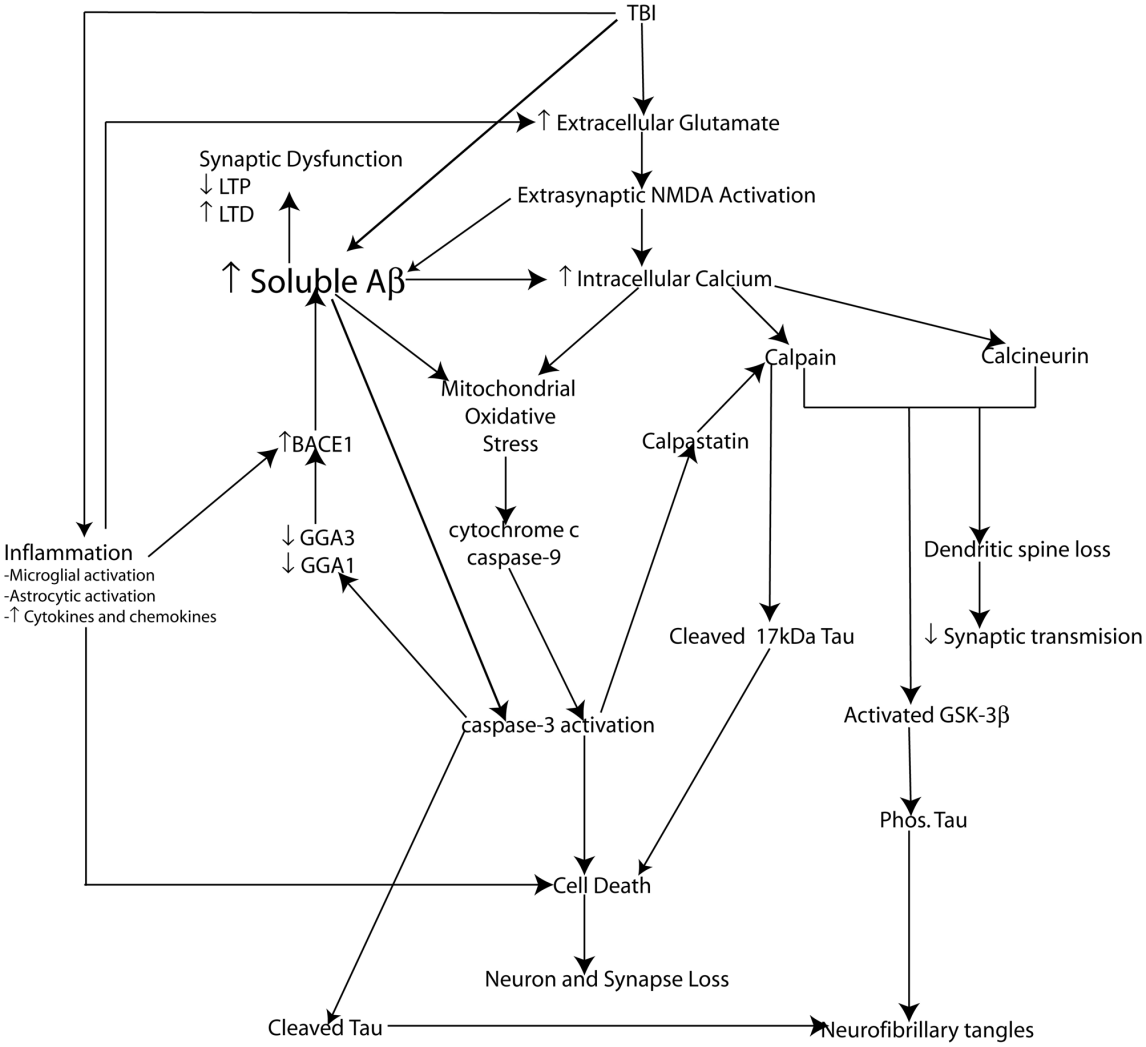
**Schematic representation of potential molecular mechanisms modulating Aβ and Tau pathology following injury.** Loss of neurons, synapses, and dendritic spines as well as elevated soluble Aβ levels, amyloid plaque deposition along with elevated phosphorylated tau levels and the formation of neurofibrillary tangles (NFT's) are pathological features common to both traumatic brain injury (TBI) and Alzheimer's disease (AD). Not surprisingly the pathophysiological mechanisms leading to cell death, synaptic dysfunction and altered Aβ and tau pathology also overlap. Glutamate mediated excitotoxicity is a key initiator of cell death following traumatic brain injury (TBI). Excessive glutamate induced activation of extrasynaptic NMDA receptors leads to dramatically elevated intracellular calcium levels. Impaired calcium homeostasis in turn initiates a number of crucial downstream cellular injury processes including mitochondrial oxidative stress as well as the activation of the calcium sensitive proteases (caspases and calpains) which have been demonstrated to induce cell death *in vitro* (and hence loss of neurons and synapses) via caspase-3 activation and the cleavage of tau into a 17 kDa toxic tau fragment. TBI induced inflammatory events including activation of microglia and astrocytes and hence increased cytokine and chemokine production plays an important role in cell death following injury. Inflammation may amplify excitotoxic cell death by reducing glial glutamate transporters leading increased glutamate at the synapse exacerbating calcium dysgregulation. Experimental evidence suggests calcium-induced activation of calpain and calcineurin induces dendritic spine remodeling and loss and hence impairs synaptic transmission. Additionally, calpain and calcinuerin have both been implicated in NFT formation via cleavage induced activation of GSK-3β leading to hyperphosphorylation of tau. TBI induces elevated levels of soluble Aβ which is capable of inducing synaptic dysfunction. β-secretase (BACE1) is a key rate limiting enzyme in the production of Aβ and we have demonstrated a molecular mechanism by caspase-3 mediated depletion of the adaptor proteins GGA1 and GGA3 modulates BACE1 levels and Aβ production following experimental TBI. Experimental evidence suggests that soluble Aβ is capable of directly mediating many of the cellular injury processes characteristic of the secondary phase of TBI and hence elevated Aβ levels may be key initiating events in the development of a “feed forward” toxic cascade that links TBI to chronic neurodegeneration.

The cellular pool of NMDA receptors activated plays an important role in Aβ production and cell fate. Activation of synaptic NMDA receptors promotes the non-amyloidogenic pathway reducing Aβ generation, upregulating ERK and CAMK pathways thereby phosphorylating the cAMP responsive element protein (CREB) promoting LTP as well as the upregulation of the prosurvival gene BDNF. Conversely, activation of extrasynaptic NMDA receptors promotes the amyloidogenic pathway leading to Aβ production and has been shown to downregulate phosphorylation of CREB and enhance LTD, while also inducing a loss of mitochondrial membrane potential signaling activation of cell death pathways (Hardingham et al., 2002; Bordji et al., 2010) (Figure 4).

Elevated synaptic glutamate plays an important role in oligomeric Aβ-mediated synaptic dysfunction (impairment of LTP) and synaptic degeneration (see Revett et al., 2013). Impairments in astrocytic glutamate uptake have been identified following TBI and in AD brains and may be responsible for the increased synaptic glutamate that triggers NMDA-mediated excitotoxicity. There is a transient reduction in glial glutamate transporters GLAST (EAAT1) and GLT-1 (EAAT2) and the astrocytic splice variant GLT-1v (EAAT2b) in rodents (Rao et al., 1998; Yi and Hazell, 2006) and humans (van Landeghem et al., 2006) following TBI. These glutamate transporters handle the bulk of extracellular glutamate uptake, resulting in increased extracellular glutamate levels that contribute to excessive NMDA receptor activation and excitotoxicity. EAAT2 and EAAT2b levels are dramatically down regulated in the brains of AD patients (Scott et al., 2011). In one study the presence of novel splice variants of EAAT2 that had been shown to be non-functional in *in-vitro* assays correlated with neuronal loss in AD brains (Masliah et al., 1996; Li et al., 1997; Scott et al., 2011). Additionally, Aβ oligomers have been shown to dramatically reduce the expression of EAAT2 in astrocytic cultures (Abdul et al., 2009) and there is evidence that inflammatory cytokines such as TNFα can inhibit astrocytic glutamate transporters (see Pickering et al., 2005). Aβ or inflammation-mediated impairments in glial glutamate uptake may be a mechanism contributing to chronic activation of NMDA receptors following TBI and in AD. To this end a recent study by Li et al. (2011b) has demonstrated that Aβ oligomers can interrupt glutamate reuptake resulting in increased extracellular glutamate which is capable of triggering extrasynaptic NR2B NMDA receptor activity. This leads to inhibition of LTP and enhancement of LTD through calpain and P38 MAPK pathway activation and decreased CREB phosphorylation (Li et al., 2011b). The importance of the activation of the extrasynaptic pool of NMDA receptors to Aβ mediated excitoxicity and cognitive impairment could explain an important reason why the success of NMDA antagonism in ameliorating cognitive dysfunction post-TBI in rodents and AD transgenic mouse models could not be replicated in human TBI patients and AD sufferers. Memantine, the only FDA approved NMDA antagonist for the treatment of dementia, primarily targets the pool of extrasynaptic NMDA receptors, thereby allowing the beneficial effects of synaptic NMDA activation to remain largely unaffected (Xia et al., 2010).

## Mitochondrial dysfunction/oxidative stress in pathophysiology of TBI and AD

Impaired intracellular calcium homeostasis and the resultant mitochondrial dysfunction and oxidative stress are pathophysiological hallmarks of both TBI and AD. Mitochondrial oxidative stress occurs early in AD progression and has been extensively demonstrated in AD patients and AD transgenic mouse models (see Ansari and Scheff, 2010; Dragicevic et al., 2010; Reddy et al., 2010). Traditionally, the calcium dysregulation occurring in AD patients has been attributed to calcium loading due to ER-stress and has been linked to familial mutations in the *PSEN1* gene (see Supnet and Bezprozvanny, 2010). However, it has been shown that Aβ oligomers can themselves dramatically alter intracellular calcium levels (see Reese and Taglialatela, 2011). *In vitro* studies in rat cortical neurons have shown that Aβ can increase intracellular calcium levels via NMDA and AMPAR activation leading to mitochondrial calcium overload. As is the case post-TBI, this mitochondrial calcium overload triggers the opening of the mPTP, resulting in loss of mitochondrial membrane potential triggering ROS production and oxidative damage (Alberdi et al., 2010) (Figure 4).

In addition to activating cell death pathways, oxidative stress results in impaired functioning and transport of mitochondria from the cell body to the synapse, decreasing synaptic function and ultimately leading to neurodegeneration (Reddy and Beal, 2008). Aβ appears to play a direct role in mediating mitochondrial dysfunction and oxidative stress, as Aβ has been isolated from mitochondrial membranes in human AD brains and transgenic AD mouse models (see Reddy and Beal, 2008). While several studies have isolated Aβ directly from synaptosomal fractions of brain mitochondria, indicating that it may play a direct role in mitochondrial dysfunction that leads to synaptic damage (see Reddy and Beal, 2008). Mitochondrial dysfunction and oxidative stress appear to be important mechanisms affecting cognitive outcome following TBI and in AD, the evidence suggests that Aβ could directly mediate these secondary injury events resulting in neurodegeneration (Figure 4).

## Calcium dysregulation initiates phosphatase and protease activation following TBI and in AD brains:

### Calcineurin activation CaN modulate Aβ and tau pathology

CaN is a calcium regulated phosphatase that has been shown to be upregulated following TBI (Kurz et al., 2005a,b) and in AD brains particularly in astrocytes surrounding Aβ plaques (Liu et al., 2005; Wu et al., 2010). Under normal conditions CaN plays an important role in regulating neurotransmitter release and synaptic plasticity. However, following brain injury it has been implicated in the loss of dendritic spines, impairment of learning and memory through dephosphorylation of CREB, inflammation and oxidative stress through the expression of cytokines and NOS and the induction of glial apoptosis (see Campbell et al., 2012a). Campbell et al. (2012b) demonstrated in a rodent model of lateral fluid percussion that TBI induced CaN activation results in a cofilin dependent degradation of the cytoskeleton of the dendritic spines leading to shrinkage/collapse and eventual loss. This dendritic spine degeneration could be prevented by CaN inhibition (Campbell et al., 2012b). Increased CaN activity may also play a role in the dendritic spine loss and synaptic alterations evident in the brains of AD patients (see Knobloch and Mansuy, 2008). Recently, Hudry et al. (2012) demonstrated a mechanism by which Aβ induced CaN activation and mediated dendritic spine loss and remodeling through activation of the nuclear transcription factor of activated T-cells (NFAT) (Hudry et al., 2012). Cognitive decline in AD patients has been strongly associated with the expression of NFAT isoforms in particular NFAT3 whose expression is strongly linked with inflammation (Abdul et al., 2009). The neuroinflammatory effect of CaN is mediated through dephosphorylation of NFAT which can promote astrocyte activation. The importance of this mechanism to AD pathology is revealed in a study in APP/PS1 mice in which inhibition of CaN and NFAT binding in astrocytes is sufficient to reduce glial activation and Aβ production and improve cognitive performance and synaptic function (Furman et al., 2012). Dendritic spine loss/impaired synaptic function through CaN activation provides an important mechanism for cognitive decline observed in AD patients and cognitive dysfunction following TBI.

CaN activity in AD brains has been shown to correlate with NFT's indicating that CaN activity may also play an important role in tau pathology. As the tau that composes NFT's is hyperphosphorylated and the primary role of CaN is to dephosphorylate substrates this appears counter intuitive. However, it has been proposed that as CaN is activated by the binding of calmodulin and as CaN needs to bind to tau directly in order to dephosphorylate it, calcium induced calmodulin binding could interfere with CaN's ability to bind to tau and thereby prevent tau dephosphorylation. Additionally, CaN has been implicated in playing a role in dephosphorylating and thereby activating GSK-3β. As activated GSK-3β is involved in phosphorylating tau, this provides another important mechanism by which CaN activation could lead to elevated phospho-tau levels and potentially the formation of NFT's seen in the brains of TBI and AD patients which correlates with synaptic dysfunction and cognitive impairment (see Reese and Taglialatela, 2011) (Figure 4).

### Calpain activation CaN modulate Aβ and tau pathology

Calpains are ubiquitous calcium sensitive proteases that under normal conditions are transiently activated and involved in essential functions in the cell including maintaining synaptic plasticity, protein turnover and cell signaling. However, when chronically activated due to high levels of calcium and depletion of calpastatin (via caspase-3 and calpain activation), an endogenous inhibitor of calpains, they can contribute to synaptic dysfunction and neuronal death following TBI and in AD. Calpain activation is a common pathological event in experimental models of TBI. It has been robustly demonstrated in rodents using a variety of injury techniques which give rise to both focal and diffuse injuries including: FPI, controlled cortical injury, weight drop, etc. Depending upon the type of injury distinct temporal patterns of calpain activation occur. In focal injuries activation occurs within minutes to hours, whereas it is delayed under conditions of diffuse injury. Additionally, calpain activation has been observed in human TBI subjects (see Vosler et al., 2008; Saatman et al., 2010). *In vitro* and to a lesser extent *in vivo* studies have demonstrated that calpains cleave a number of substrates, which may play key roles in cognitive dysfunction following TBI. Calpains are capable of cleaving a variety of structural proteins, receptors and channel proteins, signaling enzymes, apoptotic proteins and transcription and translation initiation factors, resulting in disruption of axonal transport, axonal degeneration, alterations in signal transduction and cell death. Calpain inhibition has been shown to be neuroprotective in a number of experimental TBI studies (for a detailed review see Vosler et al., 2008; Saatman et al., 2010).

Calcium-induced calpain activation along with reduced calpastatin levels have been observed in the brains of AD patients indicating calpain dysregulation. Evidence suggests that chronic calpain activation can influence the formation of amyloid plaques, NFT's and the synaptic pathology that is observed in AD brains (Ferreira, 2012). Aβ is able to activate calpains through excessive NMDA receptor activation (see Ferreira, 2012). Activated calpains cleave a number of neuronal proteins that are involved in AD. It is possible that calpain activation in the brains of AD patients impairs cognitive function by cleaving Protein Kinase A thereby reducing CREB phosphorylation and attenuating its activity and preventing the consolidation of short-term memory to long-term. The attenuation of CREB activity can also be mediated indirectly through calpain-mediated activation of CaN A, levels of which correlate NFT's in AD brains. Calpain has been theorized to be involved in synaptic dysfunction by impairing synaptic vesicle recycling due to cleavage of Dynamin 1, which is reduced in AD brains (for a detailed review see Ferreira, 2012).

Calpain activation may also play a role in Aβ generation. *In vivo* studies in the APP/PS1 transgenic mouse model of AD showed that calpain activation resulted in dramatically increased Aβ levels and plaque deposition and that this was most likely mediated through elevations in BACE1 level and activity (Liang et al., 2010). It may also play an important role in the hyperphosphorylation of tau by the cleavage and subsequent activation of a number of kinases involved in tau hyperphosphorylation including GSK-3β, Protein kinase C, calcium/calmodulin kinase (CamK) IIα, p35 which activates Cdk5 (see Vosler et al., 2008). Conversely, calpain inhibition through overexpression of calpastatin in the APP/PS1 transgenic mouse model was found to reduce amyloid beta plaques, prevent Tau hyperphosphorylation (by preventing activation of Cdk5) and synapse loss. Importantly, inhibition was found to prevent the calpain-induced decrease in CREB phosphorylation and improve spatial memory in the APP/PS1 mice (Liang et al., 2010). These results confirm those obtained by Trinchese et al. (2008) who showed pharmacological inhibition of calpain was able to restore normal synaptic function and improve spatial and fear associated memory in APP/PS1 mice (Trinchese et al., 2008). In addition to increasing Aβ levels and hyperphosphorylating tau, calpain activation may also contribute to AD pathology through the cleavage of Tau to generate a 17 kDa toxic tau fragment. High levels of this 17 kDa Tau fragment have been found in the brains of AD patients as well as patients with other tauopathies. While the role this Tau fragment plays in neurodegeneration is not well understood, it has been implicated in playing a role in tau aggregation and could promote the formation of NFT's (Vosler et al., 2008) (Figure 4).

### Caspase-mediated apoptosis following TBI and in AD brains

Apoptosis can occur by multiple overlapping pathways. The cysteine protease family of caspases are well characterized effectors of apoptotic cell death and hence apoptotic pathways are classified as caspase-dependent or caspase-independent (Zhang et al., 2005). Caspase-dependent apoptosis has been implicated in the synaptic dysfunction/degeneration and neuronal cell loss that are hallmarks of both TBI and AD. Increased expression of caspases and caspase activation (caspases −1, −3, −7, −8, −9 and −12) have been identified clinically (Clark et al., 1999; Zhang et al., 2003, 2006; Darwish and Amiridze, 2010) and experimentally (Keane et al., 2001; Knoblach et al., 2002; Larner et al., 2004, 2005) following TBI with involvement of both the extrinsic (TNF and Fas receptor mediated) and intrinsic (mitochondrial mediated) apoptotic pathways (Minambres et al., 2008). There is accumulating evidence implicating apoptosis and in particular caspase-mediated apoptosis in the disease progression and pathology of AD (see reviews Castro et al., 2010; Rohn, 2010). Despite early difficulties associated with identifying activated caspases in postmortem AD brain tissue, evidence has been obtained demonstrating activation of caspases in AD tissue in particular the initiator caspases −8 and −9 and the effector caspases −3 and −6 (see refs Castro et al., 2010; Rohn, 2010). Additionally a study by Pompl et al. (2003) found increased transcript levels of both death associated as well as inflammation associated caspases (−1, −2, −3, −5, −6, −7, −8 and −9) in the brains of AD patients(Pompl et al., 2003). Caspases have been demonstrated to be able to cleave two key molecules involved in AD pathology, APP, and Tau, which could facilitate the production of Aβ and the formation of NFT's (see review Castro et al., 2010). Caspase-3 activation is one of the most extensively characterized mediators of cell death following TBI in humans and experimental animal models. A study by Abrahamson and colleagues demonstrated that administration of a pan-caspase inhibitor following CCI in rodents reduced caspase-3 activation and caspase cleaved APP fragments and was associated with an inhibition of TBI induced Aβ generation (Abrahamson et al., 2006). However, the mechanism by which direct cleavage of APP leads to Aβ production following TBI has not been conclusively demonstrated. Gervais and colleagues proposed that caspase-mediated cleavage of APP at APPD720 was responsible for increased Aβ generation associated with apoptosis (LeBlanc, 1995; Barnes et al., 1998; Galli et al., 1998; Gervais et al., 1999; Guo et al., 2001; Tesco et al., 2003; Sodhi et al., 2004). In contrast, our laboratory has shown that caspase activation can lead to increased Aβ generation independently of APP cleavage in Chinese hamster ovary (CHO) cells (Tesco et al., 2003). We subsequently showed that caspase-3 cleavage of the adaptor protein GGA3 leads to increased BACE1 levels and activity and increased Aβ production following TBI in rodents (Walker et al., 2012). Our data suggest that inhibition of caspase-3 activation may be beneficial in reducing Aβ production by preventing caspase-mediated depletion of GGA3 and the subsequent accumulation of BACE1 following TBI.

Activated caspase-3 has been identified in neurons within AD brains and has been demonstrated to colocalize with NFT's and amyloid plaques (Su et al., 2001). Caspase activation has been implicated in NFT formation in AD brains via Tau cleavage. There is increasing evidence that truncation of the tau protein is an important event in promoting the aggregation of tau (see Lee and Shea, 2012). C-terminal cleaved Tau (D421) has been identified in the brains of AD patients (see Lee and Shea, 2012). It has been proposed that cleavage of tau at its C-terminus causes a conformational alteration, which along with phosphorylation contributes to the aggregation of tau into tangles (Lee and Shea, 2012). Evidence to support this idea has recently been obtained via two-photon microscopy in a tau transgenic mouse model (Tg4510), demonstrating that caspase-3 activation precedes tangle formation. Importantly, the neurons in which caspase-3 activation occurred did not die acutely but instead developed tangles (De Calignon et al., 2010). It has recently been demonstrated that caspase-3 activation is an early event in a transgenic mouse model of AD (Tg2576) that triggers synaptic dysfunction long before deposition of Aβ plaques occurs in this model. In this model caspase-3 activation resulted in a permanent activation of CaN, which triggered the dephosphorylation and removal of the post-synaptic AMPA receptor GluR1 subunit leading to impairments in glutamatergic neurotransmission, which correlated with spine degeneration in CA1 pyramidal neurons and impaired hippocampal dependent memory formation (D'Amelio et al., 2011). Additionally, activated caspase-3 is enriched in the post-synaptic density within the brains of AD patients (Louneva et al., 2008). When taken together with data from AD transgenic mouse models this indicates an important role for caspase-3 in AD related synaptic degeneration. As a result, in addition to initiating apoptosis and neuronal loss following TBI and in AD brains, caspases such as caspase-3 play important roles in synaptic dysfunction and tau pathology that correlates with cognitive impairment (Figure 4).

## Inflammation in TBI and AD

Inflammatory pathway activation has been implicated in mediating secondary injury following acute brain injury as well as in chronic neurodegeneration settings such as the brains of AD patients. Widespread activation of microglia and astrocytes particularly in the vicinity of Aβ plaques has been observed in AD brains and the severity of microglial activation has been linked to the degree of brain atrophy and cognitive decline (Cagnin et al., 2001; Parachikova et al., 2007; Ferretti et al., 2012). Activated microglia and astrocytes have also been demonstrated in a variety of AD transgenic mouse models (Heneka et al., 2005; Noble et al., 2010; Schwab et al., 2010; Ferretti et al., 2012). Importantly, inflammatory markers including activated microglia and astrocytes are detected in the brain of AD transgenic mice prior to Aβ plaque accumulation, indicating that inflammation is an important initiating event in Aβ pathology (Heneka et al., 2005; Schindowski et al., 2006; Garwood et al., 2010). The early activation of inflammation in AD disease pathogenesis explains why non-steroidal anti-inflammatories (NSAID's) have only shown beneficial effects in reducing the risk of developing dementia in patients who have not yet shown any signs of mild cognitive impairment (the prodromal state of AD).

TBI has been shown to result in rapid activation of microglia and astrocytes and is accompanied by the secretion of inflammatory cytokines and chemokines including IL-1β, IL-6, and TNF-α (Lloyd et al., 2008). Upregulation of IL-1β following experimental TBI in rodents has been shown to occur concurrently with increases in APP and has been implicated in Aβ production following injury (Ciallella et al., 2002). Inflammatory cytokines as well as activated microglia and astrocytes have been demonstrated to upregulate APP *in vitro* and in both wild-type and transgenic mice (Brugg et al., 1995). This upregulation of APP has been shown to increase Aβ levels, and *in vivo* studies using transgenic mice indicate that inflammation could mediate its effect on Aβ production through increased BACE1 levels and activity (Sheng et al., 2003; Heneka et al., 2005; Lee et al., 2008; Ferretti et al., 2012). It has been proposed that upregulation of APP and BACE1 levels and activity by inflammatory mediators results in increased Aβ production, which in turn further activates microglia creating a vicious cycle of neurotoxic events leading to chronic neurodegeneration (Sastre et al., 2008). NFκB a key transcription factor whose expression is increased post-TBI can regulate the expression of several inflammatory markers and has been implicated in the regulation of APP and BACE1 in inflammatory settings (Ferretti et al., 2012). Evidence exists that NFκB can regulate BACE1 *in vivo*, indicating that upregulation of NFκB following TBI may be one potential mechanism by which APP and BACE1 and hence Aβ increases are regulated post-TBI (Figure 4).

The vast majority of research has focused on the role of microglial activation in chronic neuroinflammation in the AD brain and following TBI. While activated astrocytes have been shown to play an important role in AD pathology in mouse models, their role in TBI induced chronic neuroinflammation has received less attention. This is due in part to the complex dual neuroprotective and neurotoxic nature of astrocytic activation following TBI (see review Laird et al., 2008). Interestingly, a recent study has shown that loss of activity of the dopamine receptor DR2 (DRD2) in astrocytes leads to astrocytic activation and chronic neuroinflammation (Shao et al., 2012). DRD2 activity regulates the expression of the small heat shock protein αB-crystallin which is neuroprotective and anti-inflammatory. A polymorphism within the ANKK1/DRD2 region has been shown to reduce the expression of DRD2 and is correlated with impaired cognition post-TBI in humans (McAllister et al., 2008). While the role of dopamine in inflammation following TBI is not well understood this new data highlighting the role of DRD2 in mediating inflammation may help to explain not only the beneficial effects of DR2 agonists on cognitive performance following experimental TBI but also the cognitive impairments witnessed following administration of certain antipsychotics such as Haloperidol which have been found to antagonize DRD2 (Wilson et al., 2003). Chronic astrocytic activation has important implications for Aβ generation in AD brains and following TBI. Traditionally, neurons are thought of as the major sources of Aβ generation, however, activated astrocytes may be an important source of Aβ under conditions of stress such as cerebral injury. A recent study by Zhao et al. (2011) demonstrates that the cytokines TNFα and IFNγ which are secreted under inflammatory conditions such as TBI are capable of increasing APP and BACE1 levels and increasing β-secretase processing of APP generating increased Aβ in activated astrocytes. Additionally, they demonstrated that oligomeric and fibrillar Aβ_42_ is also capable of increasing BACE1, APP and APP processing suggesting a feed forward mechanism driven by inflammation and Aβ_42_. This is a potentially important source of Aβ under inflammatory conditions as astrocytes outnumber neurons 5-fold indicating a large source of Aβ that may initiate downstream neurotoxic cascades (Zhao et al., 2011).

As is the case in AD brains inflammation is one of the earliest pathways activated post-TBI and is also one of the most persistent. In animal models of TBI inflammation has been shown to persist for at least 1yr post injury (Acosta et al., 2013). While, chronic neuroinflammation and microglial activation have been demonstrated in human TBI patients up to 17 years post moderate to severe TBI injury (Ramlackhansingh et al., 2011). The development of chronic neuroinflammation following an acute brain injury indicates that inflammatory mechanisms have the potential to exacerbate Aβ production for prolonged periods following injury and provides an important link to chronic neurodegeneration.

## TBI and chronic traumatic encephalopathy

The increased risk of developing AD following TBI appears to be associated with single events of moderate to severe injury intensities. In comparison, CTE is a neurodegenerative disease that appears to be associated with repetitive mild trauma. Originally described in 1928 in boxers and termed *dementia pugilistica* (Martland, 1928), the term CTE was coined in 1973 to describe this neuropathological entity and to encompass those who developed it as a result of playing contact sports (Corsellis et al., 1973).

CTE usually presents in mid-life and the early clinical manifestations of CTE affect behavior including increased levels of anger and irritability along with apathy. Cognitive dysfunction presents with poor episodic memory and higher order executive functioning. The late stages of the disease are characterized by movement and speech disorders. The lack of development of full-blown dementia in the majority of CTE sufferers has been attributed to the high suicide rates, which are a prominent feature of the disorder (Gavett et al., 2011). CTE is considered to be the signature injury pathology sustained in contact sports in the form of concussion and sub-concussion, but has also been identified in military personnel subjected to blast injury. Interestingly, in contrast to the model of repetitive trauma over time put forward for CTE, researchers have been able to replicate CTE pathology in mice with a single a blast exposure (Goldstein et al., 2012).

CTE is defined as a progressive tauopathy with TDP-43 proteinopathy. Extensive tau immunoreactive NFT, neuropil neuritis (NT), and glial tangles (GT) are found in the frontal and temporal cortices. While the Tau detected in CTE biopsies is hyperphosphorylated as occurs in AD and other tauopathies its anatomical distribution is unique to CTE (Stern et al., 2011). TDP-43 inclusions are widespread in CTE cases, a small subset of CTE patients with TDP-43 inclusions in the anterior horn of the spinal cord and motor cortex develop a progressive motor neuron disease, providing further evidence that some cases of ALS may be associated with TBI (McKee et al., 2010; Stern et al., 2011). However, despite the generally accepted view that the Tau accumulation observed in CTE occurs largely in the absence of Aβ, many pathological studies have revealed the presence of significant Aβ deposits in CTE cases. It has been hypothesized that the presence of the APOε4 allele may be responsible for modulating the pathology and hence the observed Aβ in some cases of CTE (Dekosky et al., 2010). Like AD, the presence of APOε4 appears to be an important genetic risk factor the development of CTE (see Lakhan and Kirchgessner, 2012).

While the pathology of CTE has been well described, little is known about the mechanisms by which, repetitive head trauma can lead to chronic neurodegeneration. This has been in part to due to the focus of most experimental TBI modeling on single events of mild, moderate or severe TBI rather than repetitive mild TBI events, and also due to the lack of appropriate models of repetitive mild TBI that encompass the rotational as well as linear acceleration/deceleration forces that appear to be important in repetitive TBI pathogenesis (see Viano et al., 2009). Newly described repetitive TBI models utilizing rodents have been identified which recapitulate many of the pathological hallmarks of repetitive TBI injuries in humans and may facilitate the study of mechanisms responsible for CTE (Shitaka et al., 2011; Kane et al., 2012; Mouzon et al., 2012; Ojo et al., 2013). Recently, Blaylock and Maroon (2011) published an interesting review proposing that the mechanism of immunoexcitoxicity may play a key role in mediating the development of CTE following repetitive TBI. This theory proposes that the interaction between the immune receptors of the CNS and the excitatory glutamate receptors that triggers a series of injury events including: ROS/rNOS production, accumulation of lipid peroxidation products and prostaglandin activation, which then leads to dendritic retraction, synaptic injury, damage to microtubules and mitochondrial suppression (see Blaylock and Maroon, 2011, for a detailed review). Presumably, the mechanisms previously described that modulate tau pathology in reference to AD such as CaN and calpain activation would apply in the setting of CTE. However, as previously mentioned while the pathology of CTE has been thoroughly described, the lack of appropriate experimental models has somewhat hampered the investigation of the molecular mechanisms. The recent development of rodent models of repetitive injury that recapitulate the pathology seen in human TBI patients should greatly increase our understanding of the mechanisms leading to CTE pathology.

## Cognitive reserve as a risk modifier for the development of dementia following TBI

**Figure 5:**
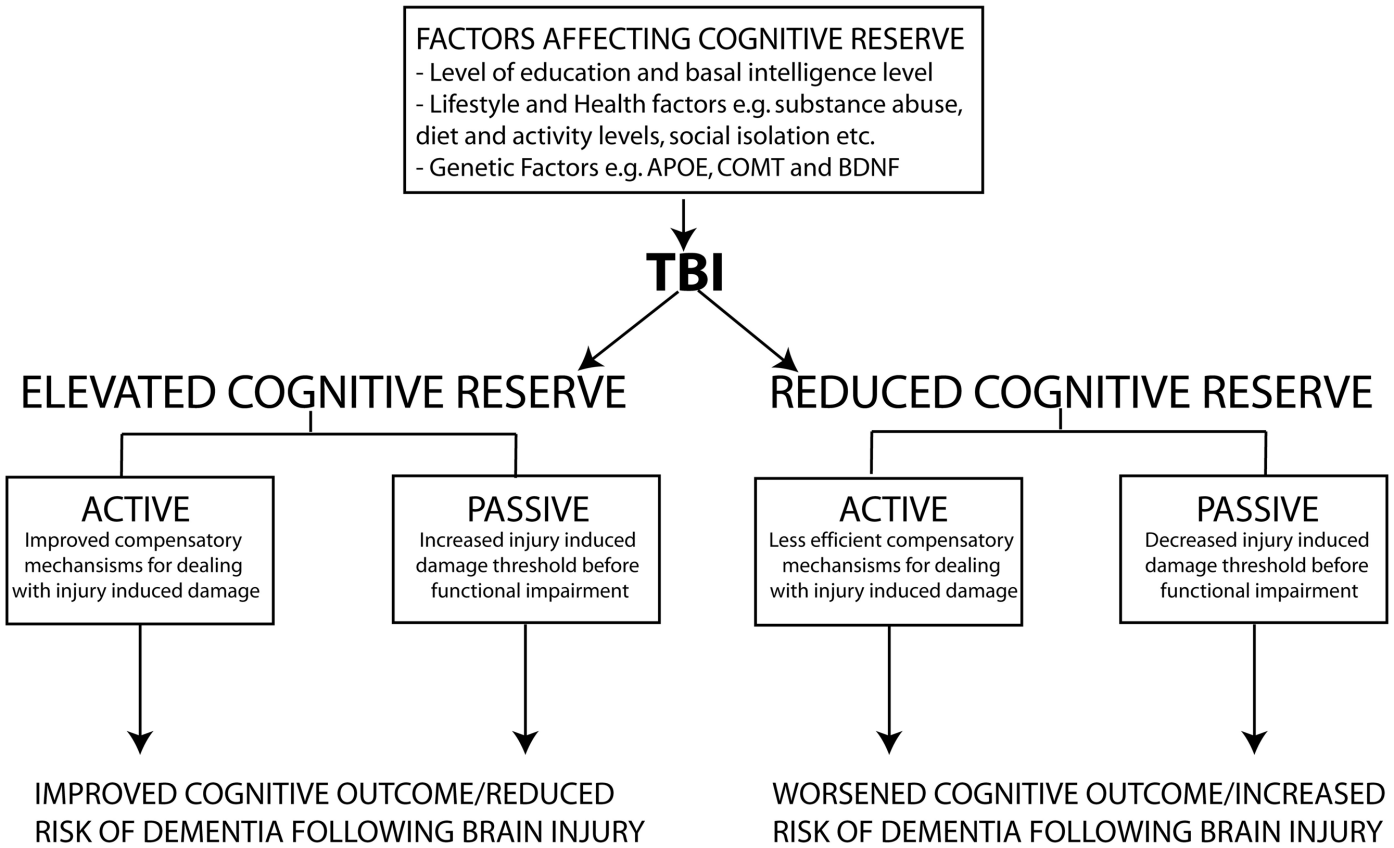
**Cognitive reserve as a risk modifier for cognitive dysfunction and dementia following TBI.** A number of environmental and genetic factors determine a persons level of cognitive reserve, those individuals with higher cognitive reserve are predicted to have a better cognitive outcome and a reduced risk of developing dementia following TBI. Individuals with lower cognitive reserve are predicted to have a poorer cognitive outcome and an increased risk of developing dementia following TBI. Cognitive reserve can be viewed as either an active (inherent compensatory mechanisms) or a passive (level of damage that can be tolerated by brain) process.

It is clear there are significant overlaps between the molecular pathways including known genetic risk factors such as APOε4 that lead to cognitive dysfunction following TBI and in neurodegenerative diseases such as AD (Tables 1, 2). However, there still remains the question of why only certain individuals with these risk factors develop dementia following a brain injury. The idea of “cognitive reserve” has been suggested to explain this discrepancy. Cognitive reserve can be looked at as either a passive or active process. A passive cognitive reserve process is where a certain amount of damage can be sustained before reaching a threshold level, which results in symptoms. While an active cognitive reserve process involves the brain actively attempting to compensate for the damage (see for a detailed review Stern, 2002). TBI is thought to accelerate the effects of aging by reducing cognitive reserve. Individuals with a greater cognitive reserve are better able to cope with the deleterious effects of a brain injury over the course of aging either through active or passive processes, whereas, those people with less cognitive reserve cannot. In view of the repetitive nature of many TBI's it is possible that a reduction in cognitive reserve following the first injury can lead to the brain becoming “primed” and any further insults to their brain may amplify the deleterious effects of the original injury. These further insults could encompass additional mild TBI's or even lifestyle and environmental exposures. Lifestyle factors such as poor health and wellbeing due to poor diet leading to increased cholesterol levels, a sedentary lifestyle, substance abuse etc may all contribute to the deleterious effects of a brain injury (see Moretti et al., 2012). Studies have shown that level of education and basal intelligence level prior to injury are strongly associated with a protective effect following injury. Additionally cognitive rehabilitation strategies employed following TBI may protect against the risk of dementia (see Moretti et al., 2012). Genetic factors (polymorphisms in a wide variety of genes) can also play an important role in cognitive reserve. While these studies are still at a relatively early stage, polymorphisms have been identified in genes that are important for modulating inflammatory responses, synaptic plasticity, and repair mechanisms as well as catecholaminergic and cholinergic function following neurotrauma (McAllister, 2010) (see review Weaver et al., 2012). Of those studied so far in TBI patients polymorphisms in Apolipoprotein E (APOE), catechol-o-methyl transferase (COMT) and brain derived neurotrophic factor (BDNF) genes have shown a significant correlation with cognitive outcome following TBI (McAllister, 2010). Further work needs to be performed to identify genetic variants involved in the injury cascades that predispose patients to a worse outcome following TBI that could predispose them to later development of dementia (Figure 5).

**Table 2:** **Common pathophysiological mechanisms in TBI and AD**.

**Molecular dysfunction**	**TBI (clinical and experimental)**	**AD (human brain and transgenic mouse model)**
Impaired synaptic plasticity ↓LTP ↑LTD	Albensi et al., 2000; Scheff et al., 2005	Oddo et al., 2003; Trinchese et al., 2004
Impaired glutamate transport ↓GLAST/EAAT1 ↓GLT-1/EAAT2	Rao et al., 1998; van Landeghem et al., 2006; Yi and Hazell, 2006	Masliah et al., 1996; Li et al., 1997; Scott et al., 2011
↑NMDA receptor activation	Palmer et al., 1993	Revett et al., 2013
Intracellular calcium dysregulation	Sun et al., 2008; Saatman et al., 2010	See review Supnet and Bezprozvanny, 2010
Mitochondrial dysfunction	Xiong et al., 1997; Gilmer et al., 2009	Dragicevic et al., 2010; Reddy et al., 2010
Oxidative stress	Ansari et al., 2008; Cheng et al., 2012	Ansari and Scheff, 2010
Calcineurin activation	Kurz et al., 2005a,b	Liu et al., 2005; Wu et al., 2010
Calpain activation	Vosler et al., 2008; Saatman et al., 2010	Trinchese et al., 2008; Ferreira, 2012
Apoptosis	Minambres et al., 2008; Stoica and Faden, 2010	Castro et al., 2010; Rohn, 2010
Caspase-3 activation	Clark et al., 2000; Stone et al., 2002; Abrahamson et al., 2006; Walker et al., 2012	Tesco et al., 2007; see review Castro et al., 2010; Rohn, 2010
↑BACE1 level and activity	Blasko et al., 2004; Loane et al., 2009; Walker et al., 2012	Fukumoto et al., 2002; Holsinger et al., 2002; Sun et al., 2002; Tyler et al., 2002; Tesco et al., 2007; Cole and Vassar, 2008
↓GGA3 and GGA1 adaptor proteins	Walker et al., 2012	Tesco et al., 2007; Walker et al., 2012
Inflammation and chronic microglial activation	Ramlackhansingh et al., 2011; Acosta et al., 2013	Cagnin et al., 2001; Heneka et al., 2005; Schindowski et al., 2006; Parachikova et al., 2007; Garwood et al., 2010; Ferretti et al., 2012

## Conclusions and future directions

It is clear that there is significant overlap between the secondary injury mechanisms and pathology observed following TBI and in the brains of AD patients. These common mechanisms include altered glutamatergic signaling, calcium dysregulation, inflammation, mitochondrial oxidative stress, and apoptosis. Importantly, it has been demonstrated that many of these processes remain activated for prolonged periods of time following injury, providing an important link between the acute injury and the persistent cognitive impairments that have been observed following TBI. Significant experimental evidence from *in vitro* studies an *in vivo* animal models suggests that the activation of these biochemical pathways could potentially modulate Aβ and tau pathology. TBI has been demonstrated to lead to increased Aβ production and there is evidence that the injury induced modulation of BACE1 may play a key role in this increase. As experimental evidence indicates that Aβ is able to directly modulate many of the neurotoxic biochemical pathways induced by TBI, we postulate that Aβ may be the important link in initiating and sustaining a vicious neurotoxic cycle that leads to chronic neurodegeneration.

However, in order to develop therapeutic interventions to prevent or reverse the cognitive dysfunction observed following TBI and in order to prevent the potential for development of dementia later in life, additional research must be performed to identify the molecular mechanisms that mediate the activation of these neurotoxic biochemical pathways. This will involve utilizing transgenic and knockout mice or the development of highly specific inhibitors that target molecular pathways of interest such as those implicated in neuro-inflammation following TBI. The need for a more thorough understanding of the molecular mechanisms linking TBI and chronic neurodegeneration is exemplified by the case of CTE. While there is evidence supporting a solid link between a history of repetitive mild TBI and the development of CTE and the pathology has been well characterized very little is known about the mechanisms that lead to this altered tau pathology. The development of new experimental animal models of repetitive TBI that more closely replicate CTE pathology along with detailed biochemical analysis of the brains of CTE patients will facilitate this.

### Conflict of interest statement

The authors declare that the research was conducted in the absence of any commercial or financial relationships that could be construed as a potential conflict of interest.
